# Altered Antioxidant System Stimulates Dielectric Barrier Discharge Plasma-Induced Cell Death for Solid Tumor Cell Treatment

**DOI:** 10.1371/journal.pone.0103349

**Published:** 2014-07-28

**Authors:** Nagendra K. Kaushik, Neha Kaushik, Daehoon Park, Eun H. Choi

**Affiliations:** Plasma Bioscience Research Center, Kwangwoon University, Seoul, Korea; University of Colorado, Denver, United States of America

## Abstract

This study reports the experimental findings and plasma delivery approach developed at the Plasma Bioscience Research Center, Korea for the assessment of antitumor activity of dielectric barrier discharge (DBD) for cancer treatment. Detailed investigation of biological effects occurring after atmospheric pressure non-thermal (APNT) plasma application during *in vitro* experiments revealed the role of reactive oxygen species (ROS) in modulation of the antioxidant defense system, cellular metabolic activity, and apoptosis induction in cancer cells. To understand basic cellular mechanisms, we investigated the effects of APNT DBD plasma on antioxidant defense against oxidative stress in various malignant cells as well as normal cells. T98G glioblastoma, SNU80 thyroid carcinoma, KB oral carcinoma and a non-malignant HEK293 embryonic human cell lines were treated with APNT DBD plasma and cellular effects due to reactive oxygen species were observed. Plasma significantly decreased the metabolic viability and clonogenicity of T98G, SNU80, KB and HEK293 cell lines. Enhanced ROS in the cells led to death via alteration of total antioxidant activity, and NADP^+^/NADPH and GSH/GSSG ratios 24 hours (h) post plasma treatment. This effect was confirmed by annexin V-FITC and propidium iodide staining. These consequences suggested that the failure of antioxidant defense machinery, with compromised redox status, might have led to sensitization of the malignant cells. These findings suggest a promising approach for solid tumor therapy by delivering a lethal dose of APNT plasma to tumor cells while sparing normal healthy tissues.

## Background

Cancer is the foremost cause of increasing human death in economically developed countries [1]. Chemotherapy [2] and photodynamic therapy [3] are frequently applied in cancer therapy to eradicate tumor cells for maximum treatment efficacy, but they also cause side effects that influence normal healthy cells. The use of radiotherapy is only 40% effective if used prior to surgery [4]. Although medical science has progressively improved treatment techniques to cure cancer, treatment approaches are still imperfect [5] due to inadequate drug distribution, dose limiting toxicity, and poor cancer cell selectivity. Nevertheless, even with many advances in chemotherapy and radiotherapy, survival rates have persistently decreased over the past years. Hence, a new cancer treatment modality is required to improve survival rates.

The use of non-thermal atmospheric-pressure plasma has recently expanded into biomedical fields (a research area called ‘plasma medicine’) [6]. Plasma sources usually contain a mixture of charged particles, radicals (e.g., reactive oxygen species (ROS)) and other reactive molecules (e.g., hydrogen peroxide, nitric oxide) as well as photons (UV). Free radicals play a big role in cellular redox signaling pathways, but high levels of ROS can have adverse effects on cells and lead to activation of cellular apoptotic pathways. Recently, our group reported valuable effects of non-thermal plasma on cancer cell death [7]. Several reports on the application of plasma for treatment of cancer were limited to a few types of cancer targets [8]–[16], which is not sufficient to establish non-thermal plasma effects on every type of cancer. Different types of cancer cell lines may have different responses to the same treatment therapies. Plasma-induced cancer cell death seems to be dependent on cellular ROS pathways [17]. Some researchers claim that ROS induced by anticancer drugs produce a shift in cellular antioxidant machinery [18], [19] and in mitochondrial membrane potential, which is related to induction of programmed cell death (apoptosis) in cancer cells [20], [21].

Herein, we report on APNT plasma interaction with three tumor cell lines, human glioblastoma cells (T98G), thyroid carcinoma cells (SNU80) and oral carcinoma cells (KB) and a non-malignant embryonic cells (HEK293). It is crucial to explore the interactions between the production of plasma-induced reactive species and cellular responses. While plasma–mediated oxidative stress may bring about harmful or beneficial cellular responses, one should examine carefully the plasma-dependent effects within target cells by comparing the effects on cancer and normal cells [22]. Previously, we reported that plasma-induced cell death in T98G brain cancer cells and have the least toxic effect on non-malignant HEK293 cells [23]. This additional study was designed to explore the role of ROS sensitive antioxidant machinery against the APNT DBD plasma induced oxidative stress in different cancer cells.

## Materials and Methods

### Human cell lines

The human cancer cell lines glioblastoma (T98G), thyroid carcinoma (SNU80), oral carcinoma (KB) and non-malignant embryonic cells (HEK293) were acquired from the KCLB (Korean Cell Line Bank, Seoul, Korea). For the plasma-cell interaction, these cells were maintained in Dulbecco's Modified Eagle Medium (Hyclone, USA) supplemented with 10% fetal bovine serum (Hyclone, USA) and 1% penicillin-streptomycin (PS) at 37°C in a humidified atmosphere of 5% CO_2_.

### Experimental device specifications and plasma treatment

Atmospheric pressure non-thermal (APNT) DBD plasma was designed and used to provide uniform treatment for biomedical purposes. Our plasma system primarily consisted of a high-voltage power supply, electrodes and dielectrics. We used this device for the treatment of human cells in an ambient air environment. For treatment, the distance was at fixed approximately 5 mm between source and sample. Throughout plasma exposure, cells were attached to the bottom of tissue culture plates and were covered in 2 mm of growth medium. Plasma was applied for 30–240 sec (s) with 80 V input voltage. A cost- effective transformer for neon light operated at 60 Hz was used for high-voltage power supply. The upper electrode is made up of silver, and down electrode facing the sample is made of stainless steel mesh. The width of the metal mesh for DBD plasma generation was approximately 80 mm, the thickness of mesh was 1 mm and the space between the two adjacent metal grids was 1 mm. They were separated by 1.8 mm-thick glass and tightly wrapped using insulating paste. The power supply was provided by slidacs, a neon trance invertor. The operational temperature of the plasma device was of 24–35°C during treatment time. Electrical power (5.7 W) was delivered to the upper and lower electrodes to produce the APNT DBD plasma. During 240 s plasma exposure, the pH of the media increased to 8.1 and the temperature of the cell culture media increased to 34.5°C (data not shown in figures). All plasma treatments were given under ambient air conditions and the room temperature recorded by infrared camera was 26°C. For APNT DBD plasma treatment, we used 5 mL of cell suspension with of 10^5^ cells/mL on the cell culture dish (SPL, Korea) and incubated cells for approximately 20–24 hours (h) to reach confluence.

### Cells viability and survival assay

For evaluation of mitochondrial viability, cells were seeded in a cell culture dish (SPL, Korea) under similar conditions as described previously (experimental device specifications section). Cells were treated with plasma for 30, 60, 120 and 240 s. A control group without plasma treatment was included in each assay. MTT [3-(4,5-dimethyl-2-thiazolyl)-2,5-diphenyl-2H-tetrazolium bromide] was used to assess cell viability. The MTT assay is a novel method of quantifying metabolically viable cells through their ability to reduce a soluble yellow tetrazolium salt to blue-purple formazan crystals [24]. After incubation times of 24, 48 and 72 h, 20 µL/well of MTT solution (5 mg/mL; Sigma-Aldrich, Korea) was added to each well of the 96-well plate. The absorbance was measured at 540 nm after 3 h incubation using a microplate reader (Biotek, VT, USA). All assay results are reported as percentage (%) viability, which is directly proportional to the number of metabolically active cells. Percentage (%) viability was calculated as:
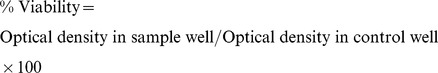



For quantitative comparison, we performed cell counts using trypan blue dye (Sigma Aldrich, Korea) and a haemocytometer.

### Tumor proliferation test by clonogenic survival assay

The clonogenic assay or colony formation assay is the most well-known cell survival assay and is based on the ability of a single cell to grow into a colony. The assay tests each cell in the population for its ability to undergo “unlimited” division. Because a single portion of seeded cells retains the capacity to produce colonies before or after treatment, cells were diluted appropriately to allow formation of colonies in 10 days. Briefly, after harvesting with 0.05% trypsin, 150–400 (depending on the exposure time) cells were plated for 20–24 h before plasma treatment in DMEM at 37°C [25]. Cultured cells were treated with plasma for 30, 60, 120 and 240 s. After plasma exposure cells were incubated in the dark in a humidified, 5% CO_2_ atmosphere at 37°C. After 8–10 days, cells were fixed with 1% crystal violet (Sigma-Aldrich, Korea) as described previously [26]. This assay provides evidence of limited tumor growth rate.

### Evaluation of cellular morphology

Scanning electron microscopic (JSM 7001F, JEOL, Tokyo, Japan) analyses were performed to examine the morphology of the cells. Briefly, after 24 h of plasma treatment, plasma exposed cells were fixed in 1 mL of Karnovsky’s fixative (2% paraformaldehyde and 2% glutaraldehyde) overnight as described in previous reports [27]. SEM sample preparation involved dehydration of the material in hexamethyldisilazane (HMDS), followed by mounting and coating on glass with carbon tape and examining using a FE-SEM. Images and cell size (length and width) were recorded with PC/SEM software (Jeol Serving Advance Technology).

### ROS detection assays

Intracellular oxidative stress ensues when an imbalance exist that favors the production of various types of ROS, such as superoxide (O_2_^•−^), hydroxyl radical (OH^•^) and hydrogen peroxide (H_2_O_2_) over antioxidant defenses [28]. To explore intracellular reactive oxygen species (ROS), we used two types of approaches. In the first scheme, fluorochrome probe 2′,7′-dichlorodihydrofluorescein diacetate (H_2_DCFDA; Invitrogen, USA) was used to detect total ROS. Briefly, after APNT plasma treatment, cells were incubated with 10 µM of H_2_DCFDA for 30 minutes (min) at 37°C in the dark. ROS fluorescence was measured using a microplate reader (Biotek, VT, USA) with excitation at 485 nm and emission at 528 nm.

In the second scheme, to ensure that the cell death upon exposure to plasma was connected with intracellular H_2_O_2_ generation, the level of intracellular H_2_O_2_ in cells was evaluated. H_2_O_2_ is highly stable and has strong oxidizing capacity and is therefore considered a strong ROS. Generally, superoxide radical can react with ambient water to form H_2_O_2_. Consequently, it can damage mitochondrial cellular components and cause cell death. ADHP (10-acetyl-3,7-dihydroxyphenoxazine) was used for H_2_O_2_ detection. ADHP reacts with H_2_O_2_ to produce highly fluorescent resorufin. All steps of detection were performed according to the manufacturer’s (Hydrogen Peroxide Cell-Based Assay Kit; Cayman chemicals, USA) instructions. Resorufin fluorescence was measured using microplate reader (Biotek, VT, USA) with excitation at 540 nm and emission at 600 nm.

### Total glutathione assay

Reduced glutathione (GSH) and oxidized glutathione (GSSG) plays key roles in cellular redox systems. It is essential to examine intracellular glutathione levels in experiments, because fluctuations in the GSH/GSSG ratio are related to human disease therapy, aging and other cell signaling activities. The level of GSSG reflects cell health and oxidative stress. Briefly, 24 h after plasma treatment, cells were washed with PBS and total cell extract was prepared separately for GSH and GSSG quantification in the lysis reagent provided with the kit. GSH, GSSG and GSH/GSSG were determined by a luminescence based biochemical method using the GSH/GSSG-Glo Assay Kit (Promega, Korea) following the manufacturer’s instructions. Luminescence was detected using a microplate reader (Biotek, VT, USA).

### NADP^+^/NADPH quantification assay

NADPH is intricately involved in protecting against ROS toxicity, allowing the renewal of GSH. Nicotinamide adenine dinucleotide phosphate (NADP) is an enzymatic cofactor involved in many redox reactions where it cycles between the reduced (NADPH) and oxidized (NADP) forms. Cell samples were prepared 24 h after treatment with plasma according to manufacturer’s instructions provided in the NADP/NADPH Quantification Kit (Sigma-Aldrich, Korea). Sample supernatants were collected and analyzed immediately for NADP^+^ and NADPH. There was no need to purify samples because this assay is specific for NADPH and NADP^+^. NADP_total_ and NADPH were quantified at 450 nm using a microplate reader (Biotek, VT, USA).

### Assessment of cell antioxidant activity

Mammalian cells have developed complex antioxidant systems to stabilize ROS and to reduce injury. Samples of cells 24 h after plasma treatment were prepared according to the manufacturer’s instructions provided in the Antioxidant Assay Kit (Cayman Chemicals, USA) and analyzed for antioxidant activity. Absorbance was detected using a microplate reader (Biotek, VT, USA) at 405 or 750 nm.

### Caspase 3/7 activity assay

Caspases, a family of cysteine proteases, are the central regulators of apoptosis. Caspases are also necessary for other biological purposes, including cell proliferation, and differentiation. The most studied members of this cysteine protease family include executioner caspase-3 and caspase-7, which play a central role in cell apoptosis and differentiation [29]. To measure apoptosis, the Caspase-Glo 3/7 Assay Kit (Promega, Korea) was used according to the manufacturer’s instructions. Luminescence was measured using a luminometer (Biotek, VT, USA).

### Mitochondrial membrane potential (ΔΨm) analysis

Mitochondria serve a major role in cell apoptosis induced by many stimulating factors, and the drop of mitochondrial membrane potential (ΔΨm) is an earlier event during apoptosis [30]. The mitochondrial membrane potential was monitored by Mito Flow (Cell Technology Inc, USA). The Mito Flow assay utilizes a cationic dye to visualize the change of mitochondrial membrane potential (MMP). It is a cell permeable, rhodamine-based dye. Membrane potential driven accumulation of the dye within the inner membrane region of healthy functioning mitochondria results in a strong red-orange fluorescence. In the apoptotic cells the dye does not accumulate in the mitochondria, therefore these cells exhibit a lower fluorescence signal. A total of 2×10^5^ cells/mL were treated with the plasma for 0, 30, 60, 120 and 240 s. 5 µL of 20X MitoFlow dye was added before the cells were harvested and incubated for 30 min at 37°C. Then, the cells were collected, rinsed with dilution buffer and analyzed for emission at 488 nm by FACS analysis (BD FACSVerse, NJ, USA instrument and FACS suite software).

### Cell apoptosis assay (Annexin-V and PI staining)

Apoptosis is a key method by which cancer cells die after treatment [31]. To evaluate plasma induced cell death, annexin V/PI staining was performed followed by flow cytometry. Cells were seeded and treated for 120 s because this was shown to exert maximum plasma effect. Briefly, 24 h after plasma treatment, cells were collected and subjected to annexin V/PI staining using the EzWay AnnexinV-FITC Apoptosis Detection Kit (Koma Biotech Inc, Seoul, Korea) according to the manufacturer’s protocol by FACS analysis (BD FACSVerse NJ, USA). Actinomycin D (5 µg/mL, Cayman chemicals, USA) was used as a positive control reagent for apoptosis activation (data not shown).

### Statistical analyses

All result values were expressed as the mean ± standard deviation (S.D.) of four independent tests. Statistical analysis was performed using Student’s *t*-test. Statistical significance was recognized at *p<0.05 and **p<0.01.

## Results

### APNT plasma reduced cell metabolic viability and colony forming capacity

**Figure 1:**
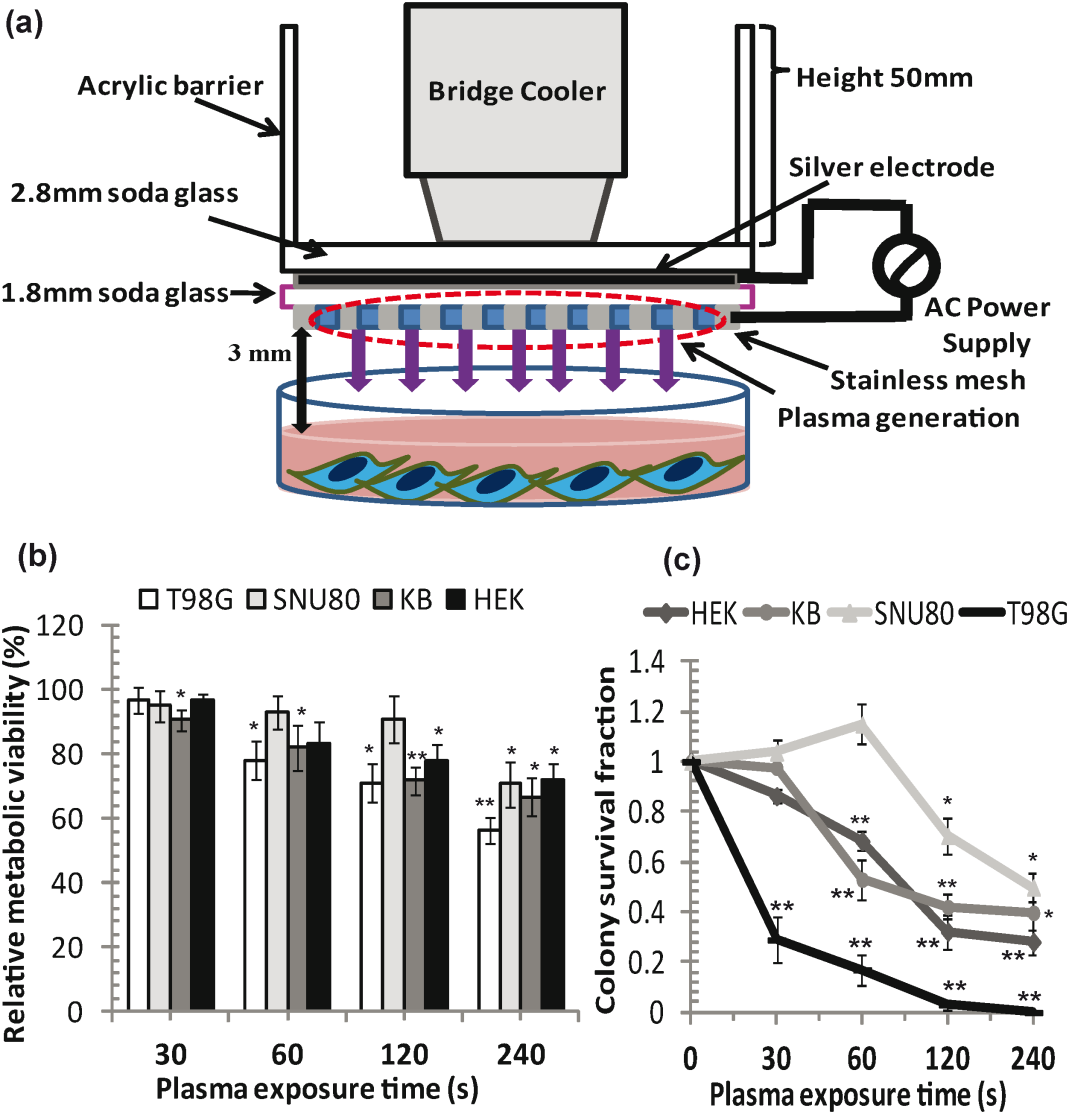
APNT DBD plasma shows growth inhibitory effect in cancer cells. (a) Schematic diagram of an atmospheric pressure non-thermal dielectric barrier discharge (APNT DBD) plasma device. (b) Metabolic viability (%) of cells after plasma treatment was compared after 24 h incubation. (c) Colony forming capacity and clonogenic survival of exponentially growing T98G, SNU80, KB and HEK293 cells. Results from four independent experiments are shown as mean ± SD, and Student’s *t*-test was performed to controls (*p<0.05 and **p<0.01).

The APNT DBD plasma device was used for cell treatment (**Figure 1a**) under conditions similar to those described previously (experimental device specifications and plasma treatment section). In our study we examined the viability of T98G glioma, SNU80 thyroid cancer, KB oral cancer and HEK293 non-malignant cells. MTT assay results indicated that the APNT plasma has a greater inhibitory effect on cancer cells than on normal cells in a dose/incubation time-dependent manner. We observed that the cells exposed to 30 and 60 s plasma treatments showed a lesser effect than those exposed to a 120 and 240 s treatments. However, plasma did not induce any inhibitory effect on SNU80 cells (*p*>0.05) at treatments as much high as 120 s. A significant inhibitory effect was seen after 60 s plasma exposure of cells, as shown by inhibition of cell viability up to 22.1% (*p*<0.05), and 17.9% (*p*<0.05) respectively in T98G, and KB cells, with a range of viability of 77.9%–83.1% (*p*<0.05). We found that after 120s exposure, up to 29%, 28.2% and 26% cells died in T98G, KB and HEK293 population, respectively, and their viability was 71% (*p*<0.05), 71.8% (*p*<0.05) and 74% (*p*<0.05), respectively, after treatment. Our data clearly show that a higher dose (240 s) of plasma exposure results in a severe decrease in metabolic activity and 56.4%–70.7% viability (*p*<0.05) of all four types of cells (**Figure 1b**).

To confirm the changes in metabolic viability of cells, we performed clonogenic assay. **Figure 1c** demonstrates the effect of the APNT plasma on the colony forming capacity. APNT plasma treatment resulted in a decline in the viability of cells, which resulted in the reduction in the number of colonies formed after plasma exposure. The colony survival fraction of T98G, KB and HEK293 cells was found to be drastically decreased and directly depended on plasma exposure time. Even after 60 s plasma exposure, a highly significant decline (*p*<0.01) in colony survival fraction up to 0.2, 0.52 and 0.68, respectively was detected in T98G, KB and HEK293 cells. SNU80 cells were not significantly affected (*p*>0.05) by a plasma treatment of up to 60 s. A significant, drastic decline in the survival fraction (*p*<0.01) was observed after a 120 s plasma treatment with a survival fraction of 0.04, 0.4 and 0.321, respectively in T98G, KB and HEK293 cells. After a 240 s plasma exposure, we found significant inhibition (*p*<0.05) in the clonogenic capacity of all four cell lines compared to untreated controls. This shows that these treatments exert an inhibitory effect on the colony formation capabilities of all cancer cells at all doses and also indicates that plasma treatment causes cell death as reflected by decreased clonogenicity.

### Survival Rate

**Figure 2:**
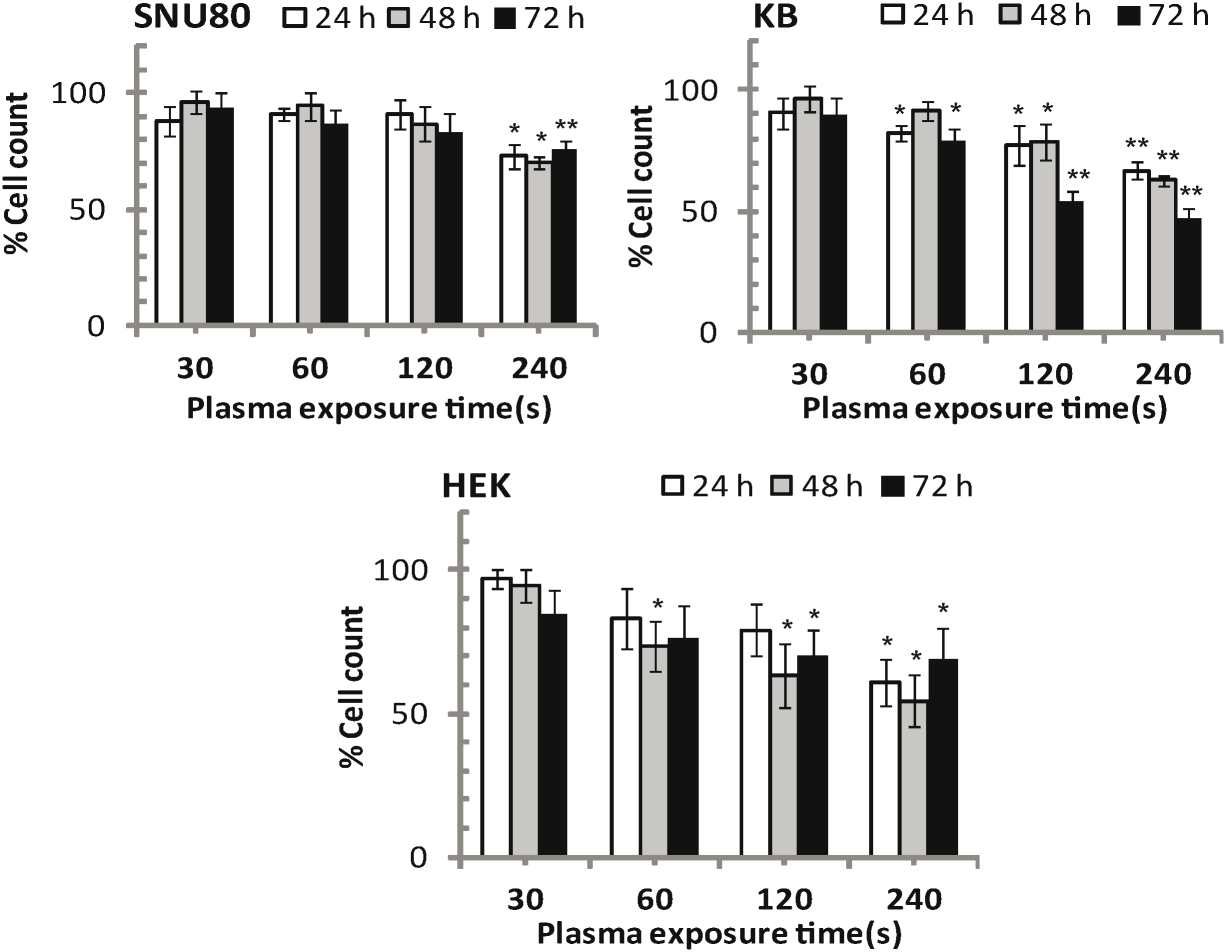
The cell counts (relative to control) showed exposure/incubation time-dependent death rate. KB cells underwent more severe loss than SNU80 and HEK293 by APNT DBD plasma treatment. Results from four independent experiments are shown as mean ± SD, and Student’s *t*-test was performed to controls (*p<0.05 and **p<0.01).

We quantitated cells by counting cell number using a hemocytometer during 3 day incubation. The ratio of test cell and control cells is shown in **Figure 2**. The cell number ratio was significantly decreased after 120 s plasma exposure for cancer cells and for non-malignant HEK293 cells. The results of this study clearly demonstrate that cell number is reduced in an exposure time dependent fashion. DBD plasma had inhibitory effect on the growth of T98G cells in an exposure time-dependent manner. Plasma treatment for 30, 60, 120 and 240 s decreased the T98G cell count to 73% (*p*<0.05), 66% (*p*<0.05), 58% (*p*<0.01), and 20% (*p*<0.01), respectively, at 72 h incubation [25]. The SNU80, KB and HEK293 cells count were significantly decreased (*p*<0.05) at a 240 s plasma-dose, but not after a 30 s plasma dose (*p*>0.05). In addition, the KB cell survival percentage was significantly decreased (*p*<0.05) after a 120 s plasma dose at all incubation time, which could be predicted from increased intracellular and extracellular ROS levels or the failure of cell antioxidant systems. Interpreted together, the results from the MTT, clonogenic, survival rate assays clearly demonstrate that the plasma sensitized cells in a dose dependent manner, which may be due to an increase in mitotic (linked to cytogenetic damage) or interphase (apoptosis) death.

**Figure 3:**
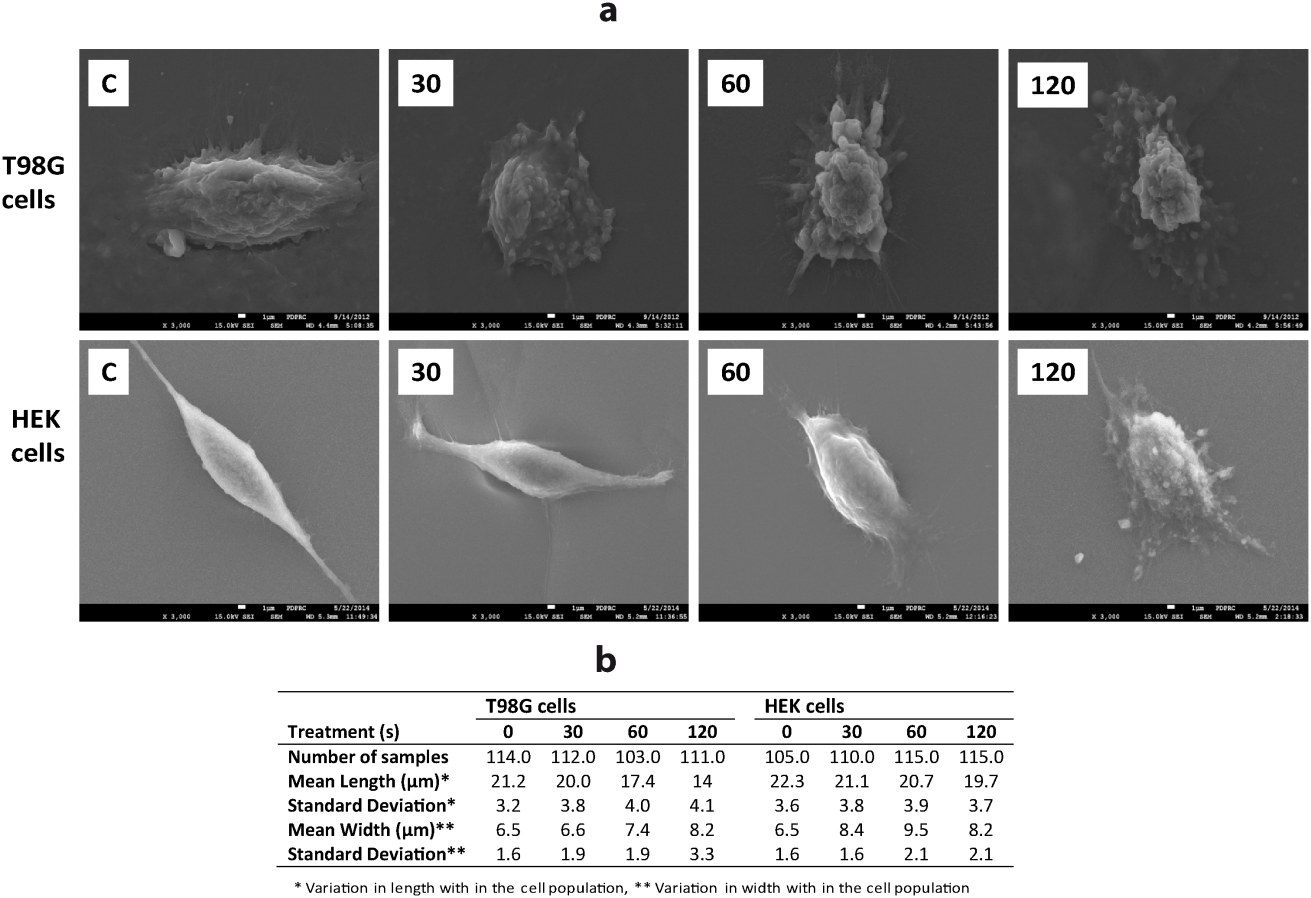
APNT plasma effects on morphological structure of T98G cancer and HEK293 cells. (a) Cell morphology analyzed by scanning electron microscope (SEM). Cells have blebbing and clear changes in morphology on their outer surface 24 h after plasma treatment. (b) Summary of cellular morphological parameters (length and width).

**Figure 4:**
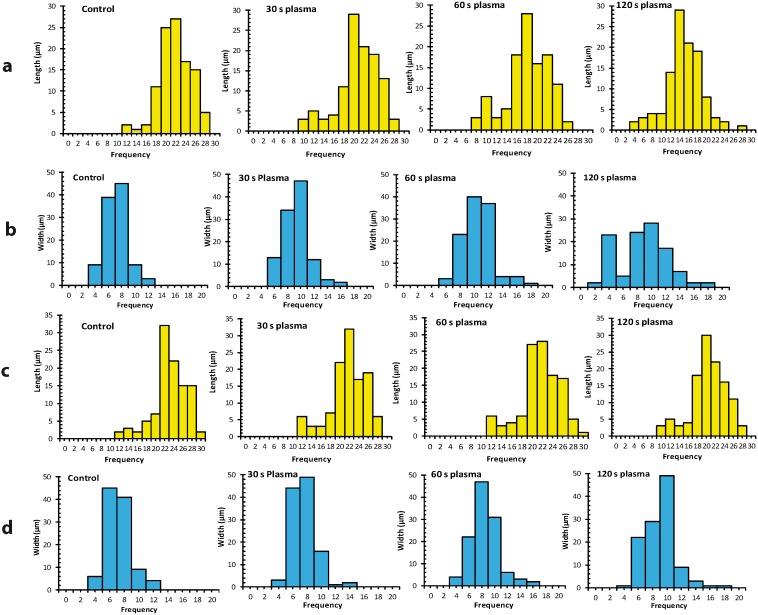
Analyses of the size variability of T98G and HEK293 cells. (a) and (b) show the frequency distribution of length and width, respectively, in the T98G cell population. (c) and (d) shows the frequency distribution of length and width, respectively, in the HEK293 cell population.

The cell morphology study also revealed that the morphology of the cells was affected by APNT plasma treatments. **Figure 3** shows the differences in morphology of the treated T98G and HEK293 cells, compared to the untreated controls. **Figure 4** shows the frequency distribution of cell size (length and width) and images of cells analyzed using the JEOL PC/SEM software system. We found significant (*p*<0.01) size variability between control and plasma treated (≥60 s dose) T98G cancer cell populations. HEK293 cells only showed significant (*p*<0.01) morphological variations at a dose of 120 s. The membrane of T98G cells membrane started blebbing and leaking inner components after a ≥60 s dose of plasma exposure and these cells ultimately died via apoptotic pathway.

### Intracellular ROS accumulation

**Figure 5:**
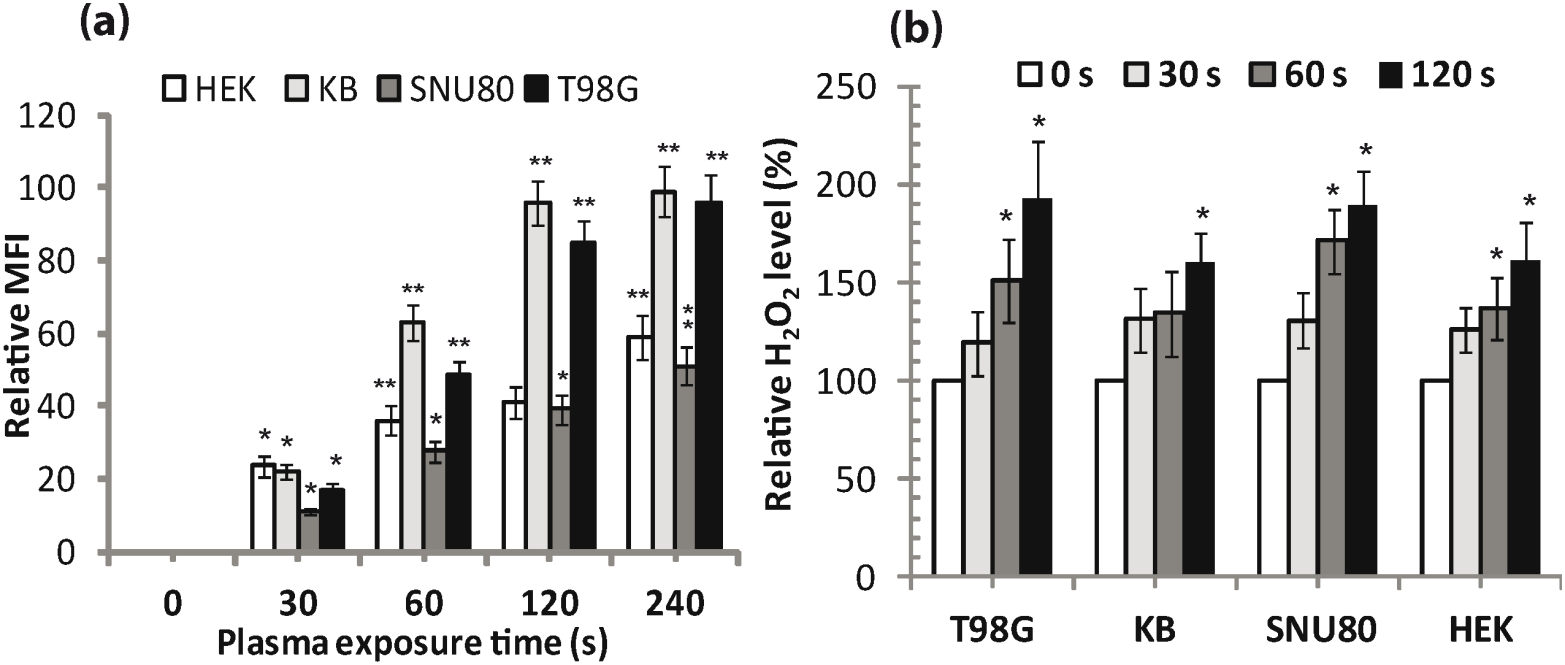
Induction of ROS in APNT DBD plasma treated cells. (a) T98G, SNU80, KB and HEK293 cells were treated with the oxidation-sensitive fluorescent probe 2,7-dichlorodihydrofluorescein diacetate (H_2_DCFDA) for detection of total ROS, (b) detection of H_2_O_2_ level (in µM) in cells observed at 24 h after exposure. In (a) and (b), all fluorescence levels were expressed as fluorescence intensity (FL intensity). Results from four independent experiments are shown as mean ± SD, and Student’s *t*-test was performed to controls (*p<0.05 and **p<0.01).

**Figure 5** shows ROS measurement in plasma exposed cells. Enhanced levels of different ROS lead to increased oxidative stress that result in DNA, lipid, and protein destruction in cells. Therefore, to determine if plasma enhanced oxidative stress, cells were labeled with the oxidation sensitive probe (H_2_DCFDA) that is capable of being oxidized to fluorescent product (DCF) by reactive oxygen species and other peroxide radicals generated from ROS. **Figure 5a** displays total ROS production in cells at 24 h following plasma treatment. Plasma increased the DCF fluorescence by 1.2 to 2-folds in cells compared with untreated controls and this increase depended on plasma dose. However, a less significant change (*p*>0.01) in ROS production was observed in cells treated with a low plasma dose (30 s) at 24 h. A 240 s plasma dose significantly increased (*p*<0.01) the intracellular ROS by 2-fold in T98G and KB cell lines. Plasma treatment of SNU80 and HEK293 cells significantly increased (*p*<0.05) the intracellular ROS by 1.2 to1.5-folds.

We further examine the levels of intracellular H_2_O_2_ in the cells as described earlier (ROS detection assays section). It is renowned that H_2_O_2_ is a cytotoxic agent whose levels must be minimized by the action of antioxidant defense system. **Figure 5b** shows increased fluorescence for H_2_O_2_ observed in cells in plasma dose-dependent fashion at 24 h. A significantly higher intracellular level of H_2_O_2_ was observed in plasma treated cancer cells than non-malignant HEK293 cells. A 60 s plasma dose significantly increased (*p*<0.05) the resorufin fluorescence in T98G, SNU80 and HEK293 cells. However, A 120 s plasma dose significantly increased (*p*<0.05) the resorufin fluorescence by 1.6 to 2-fold at in all cells compared to untreated controls.

### Instabilities occurs in cellular redox status by APNT plasma

**Figure 6:**
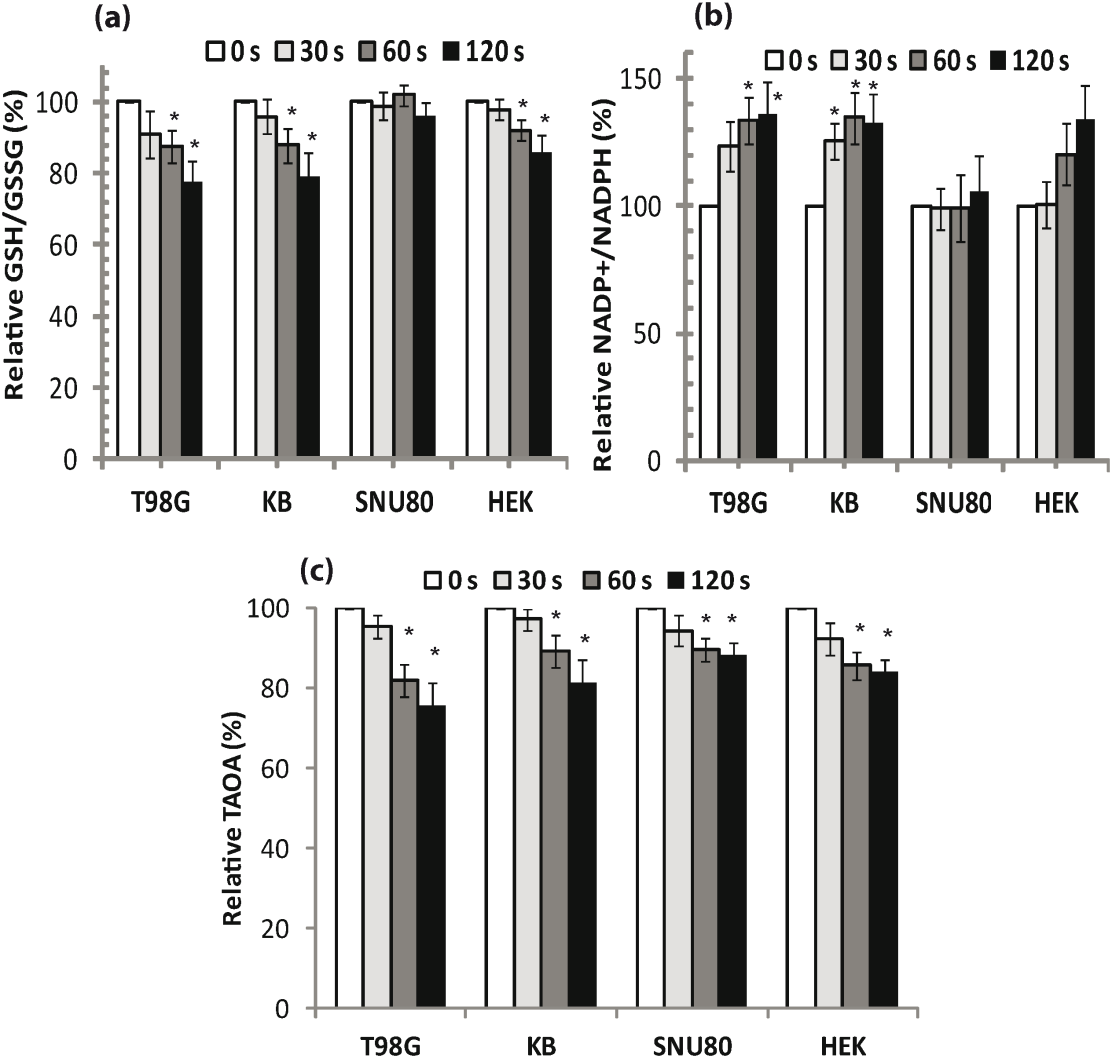
Changes in redox indicators due to APNT plasma exposure. (a) Detection of GSH/GSSG levels in cells. (b) NADP^+^/NADPH ratio in cells levels of NADP^+^ and NADPH were measured using a standard prepared for NADPH. The ratio of NADP^+^ and NADPH was plotted as a function of treatment time. (c) Total antioxidant activity (TAOA) was assessed in APNT plasma treated cells. Results from four independent experiments are shown as mean ± SD, and Student’s *t*-test was performed to controls (*p<0.05 and **p<0.01).

GSH has a central role in maintaining the cellular redox homeostasis. Some previous reports have also claimed that many diseases such as cancer, neurodegenerative disorders, cystic fibrosis, and Crohn’s disease occurs if there is a disturbance in GSH [32], [33]. We assessed total cellular glutathione (both reduced and oxidized) in our study. **Figure 6a** shows a significant decrease (*p*<0.05) in GSH and an increase in GSSG in T98G, HEK293 and KB cells, 24 h after 60 and 120 s plasma treatments. In contrast, changes in GSH and GSSG levels inside plasma treated SNU80 cells were not significant (*p*>0.05) 24 h after treatment. A reduction in GSH and a simultaneous increase in GSSG (which was more prominent after 60 and 120 s plasma treatments) suggest a compromised redox state in both T98G and KB cells. The ratio of GSH/GSSG, a measurement of redox status, was found to decrease significantly (*p*<0.05) in T98G and KB cells after 60 and 120 s plasma treatments (**Figure 6a**). The ratio of GSH/GSSG in HEK293 cells also decreased significantly only after a 60 s plasma dose. Plasma significantly decreased the level of GSH/GSSG by 22.2%, 21% and 14% in T98G, KB and HEK293 cells, respectively, after a 120 s plasma treatment.

It has been reported that NADPH is vital for GSH synthesis. Therefore, we conducted a study to determine the ratio of NADP^+^/NADPH in cells. The ability of cell to neutralize H_2_O_2_ by glutathione peroxidase (GPx) depends on the regeneration of NADPH from NADP^+^ by the GSH synthesis pathway. We found that treatment with plasma may interrupt regeneration of NADPH in cells. After 24 h, the level of NADPH was reduced significantly (>25%, *p*<0.05) in T98G and KB cells treated with plasma for 60 and 120 s plasma compared with untreated controls. In contrast, plasma treatment did not significant change this (*p*>0.05) in SNU80 and HEK293 cells.

**Figure 6b** shows that the ratio of NADP^+^/NADPH (an indicator of redox status) was increased up to 20%–35% in T98G, KB and HEK293 cell lines compared with untreated control cells 24 h after plasma treatment. Conversely, a 30 s plasma dose did not significantly change the NADP^+^/NADPH ratio (*p*>0.05) in all four cells when compared to untreated controls. No significant change in the ratio of NADP^+^/NADPH (*p*>0.05) was observed in HEK293 and SNU80 cells at up to a 120 s plasma dose. The increase in the NADP^+^/NADPH ratio clearly directs the compromised regeneration of NADPH from NADP^+^ under these experimental conditions. For this reason, failure to regenerate NADPH during treatment could lead to enhanced intracellular oxidative stress levels.

### Reduction in the antioxidant system activity in cells

Because an increase in ROS has been demonstrated in plasma exposed cells, we measured the total antioxidant events in cells. Oxidative stress ensues when antioxidant mechanisms are overwhelmed by generation of excessive reactive oxygen and nitrogen species that damage membrane lipids, proteins and nucleic acids [34]. We observed that the plasma resulted in a decrease in antioxidant activity in cells. **Figure 6c** shows a significant decrease in the endogenous antioxidant activity level (*p<*0.05) in all four type of cells after 60 and 120 s plasma doses compared with the untreated control group. However, there was no significant decrease (*p*>0.05) in antioxidant activity in cells after 30 s plasma treatment. Plasma decreased the antioxidants activity by 24.5%, 18.6%, 11.7% and 15.8% of T98G, KB, SNU80 and HEK293 cells, respectively, after 120 s plasma dose. Data from this experiment shows that these cells used their antioxidant system (both enzymatic and non-enzymatic) to reduce the load of ROS induced by the plasma treatment.

### Caspase activation and loss of mitochondrial membrane potential are involved in APNT plasma-induced apoptosis

**Figure 7:**
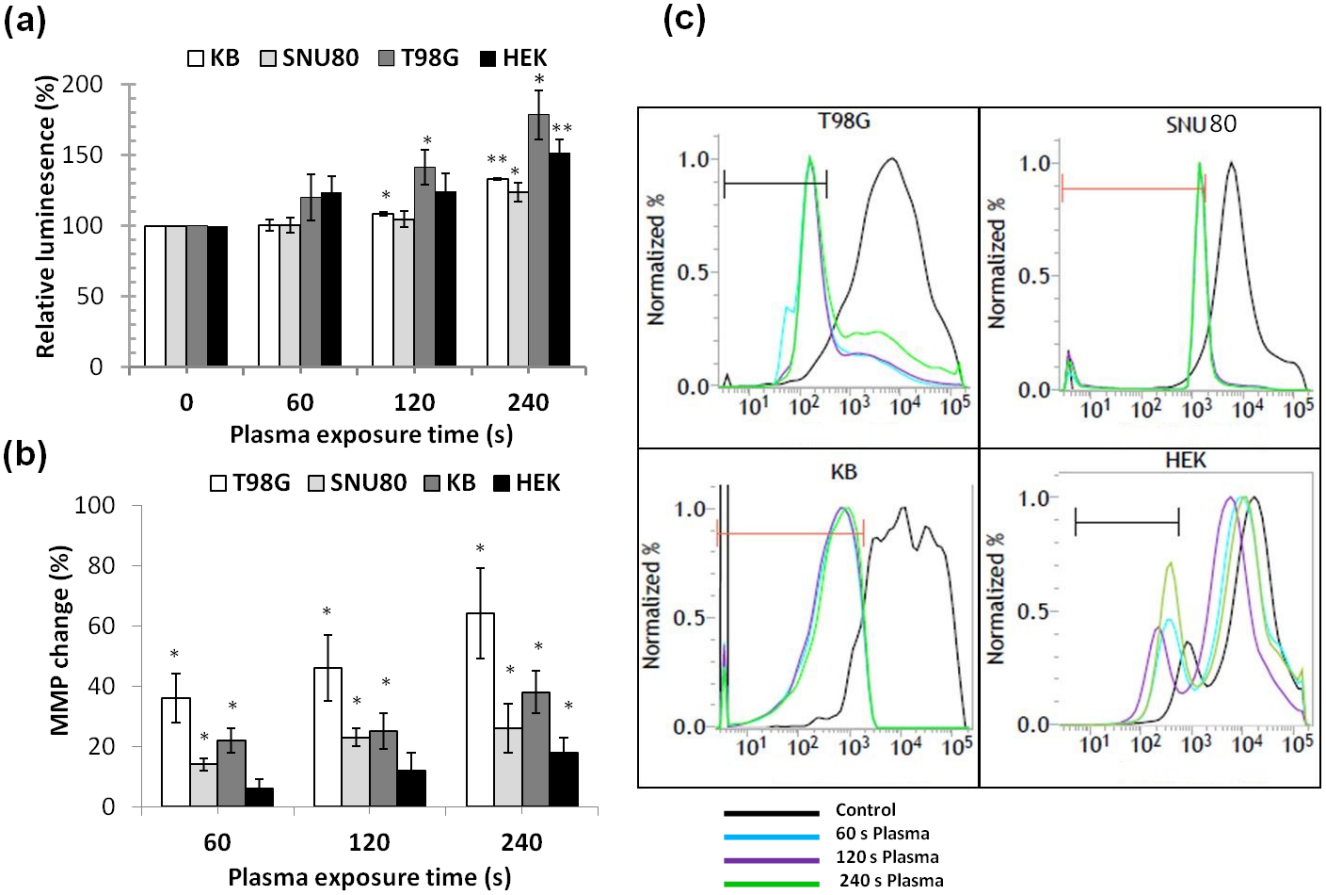
Involvement of caspase activation and loss in mitochondrial membrane potential during apoptosis. (a) APNT plasma induced activation of caspase-3/7 of human glioblastoma (T98G) and a non-malignant (HEK293) cell lines. (b) APNT plasma affects mitochondrial membrane potential of T98G, SNU80, KB and HEK293 cells. (c) Flow cytometric plot of mitochondrial membrane potential in cells, using Mito Flow rhodamine dye. Results from four independent experiments are shown as mean ± SD, and Student’s *t*-test was performed to controls (*p<0.05 and **p<0.01).

To evaluate the influence of plasma on caspase 3/7 activity, we used three cancer cell lines (T98G, KB, and SNU80) and a non-malignant cell line (HEK293). Caspase-3 and -7 play an important role in the cleavage of cellular constituents during apoptosis. **Figure 7a** shows the caspase activity in both control and plasma treated cells at 24 h post-treatment. In untreated cells, the detected level of caspase activity was related to the fraction of apoptotic cells generated in the normally growing population due to natural aging. In treated cells, caspase-3/7 activity increased over basal levels. Plasma exposure for 120 and 240 s significantly increased (*p*<0.05) the caspase -3/7 activity by 41.3% and 78.6%, respectively, in T98G cells. Plasma exposure for up to 60 s did not produce a significant (*p*>0.05) change in caspase-3/7 activity in all four cell lines. KB cells was also affected significantly (*p*<0.05) at 120 s plasma exposure, with an increase of caspase activity by 8.2%. Plasma exposure for 240 s significantly increased (*p*<0.01) the caspase activity in HEK293 cells by 52%. SNU80 plasma treated cells showed significant increases (*p*<0.05) in caspase activity only at a 240 s plasma dose. We observed a significant change (*p*<0.05) in caspase activity in all cell lines at a higher dose (240 s) of plasma.

Because mitochondria are important for both intrinsic and extrinsic apoptosis pathways, mitochondrial membrane potential was measured. An enhanced ROS level often induces a membrane permeability change, which is an early event in cell apoptosis [35]. APNT plasma generates different reactive oxygen species that can disrupt membrane polarization. Following 24 h of incubation with similar treatment conditions, cells were incubated with Mito Flow dye for 30 min and then analyzed by FACS analysis. When gated cells were examined for dye uptake, those that had been treated with plasma clearly differed in fluorescence compared with the untreated control. **Figures 7b and 7c** demonstrate the MMP change in cancer and normal cells. The MMP of 60 and 120 s plasma treated T98G, KB and SNU80 cells show significant changes (*p*<0.05) when compared to HEK293 cells. Plasma treatment for 60, 120 and 240 s induced a significant MMP change (*p*<0.05) of 36%, 46% and 64%, respectively, in T98G glioma cells (**Figure 7b**). Plasma treatment for 240 s induced significant change (*p*<0.05) in MMP of all four type of cell lines. However, plasma treatment for up to 120 s did not result in a significant (*p*>0.05) change in the MMP of HEK293 cells. **Figure 7c** shows a band shift phenomenon in cells observed after 24 h incubation. Plasma exposure resulted in membrane depolarization within the cells in a dose-dependent fashion, which is an indication of early/late apoptosis.

### Apoptosis

**Figure 8:**
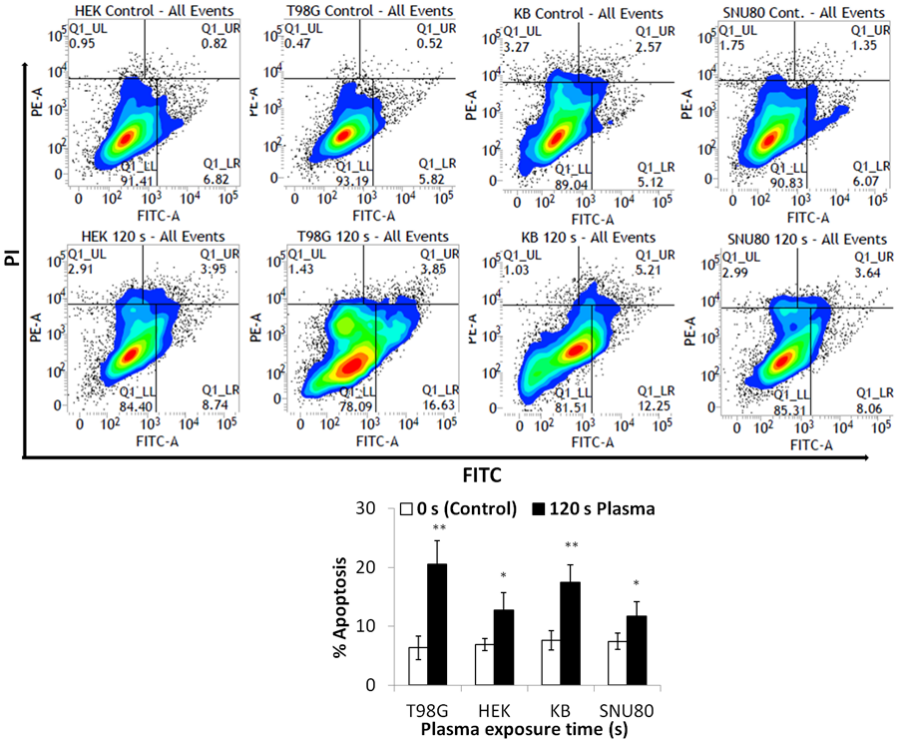
Analysis of APNT induced cell death (apoptosis). Flow cytometry data of Annexin V and PI staining of human T98G, SNU80, KB and HEK293 cells after plasma treatment. Apoptosis of each cell was evaluated after 24± SD, and Student’s *t*-test was performed to controls (*p<0.05 and **p<0.01).

Programmed cell death (apoptosis) is well known in multicellular organisms and, can be recognized by morphologic characteristics (such as membrane blebbing), cellular size and volume reduction, caspase activity, chromatin shortening etc [36]. Because cell survival was dramatically reduced in all cells and to confirm the observed MMP and caspase assay results, we next evaluated whether the plasma induced apoptosis in cells. Cell populations were evaluated by flow cytometer after staining with fluorescein isothiocyanate (FITC)-labeled annexin V (green fluorescence) and with the the nonvital dye propidium iodide (PI). This allowed discrimination between intact, early apoptotic, late apoptotic and necrotic cells [37]. **Figure 8** shows a flow cytogram with four quadrants for measuring intact, early apoptotic, late apoptotic and necrotic cells. A plasma exposure of 120 s induced significant (*p*<0.05) apoptosis in T98G, KB, SNU80 and HEK293 cells when measured 24 h after treatment. This effect was more prominent (*p*<0.01) in T98G and KB cancer cells. As shown in **Figure 8**, plasma treatment increased annexin V-FITC binding 1 to 2-fold; from the negative control of 6.34%–7.42% to 20.5% (*p*<0.01), 12.69% (*p*<0.05), 17.6% (*p*<0.01), and 11.7% (*p*<0.05) in T98G, HEK293, KB and SNU80 cell line, respectively. These results can be correlated with the results from the caspase activation and mitochondrial membrane potential change experiments.

## Discussion

Numerous reports have revealed that atmospheric non-thermal plasmas can encourage cancer cell apoptosis in a dose-dependent manner and that this can be related to DNA damage resulting from the generation of ROS [38]. Plasma action seems to be lethal for cells and modulation in the cellular metabolic activity was observed (by MTT assay) up to 24 h post-treatment. This effect was found to be dependent on the plasma dose. Current results of anticancer studies clearly demonstrate that APNT DBD plasma treatments sensitize cells by increasing cell death and failure of endogenous antioxidant system to counteract ROS burden in treated cells. In this study, we have demonstrated through the evaluation of metabolic viability, clonogenicity, cell count, ROS, and H_2_O_2_ that plasma exposure has a significant inhibitory effect on cancer cell growth, and it generated a large amount of oxidative stress in cells.Our results confirmed that intracellular ROS level was increased significantly by APNT DBD plasma treatment. The increased oxidative stress level and a resulting loss of MMP were reported to be distinctive phenomena during mitochondria-dependent apoptosis [39]. MMP loss also induces apoptosis by causing the release of cytochrome c from the inner mitochondrial space to the cytosol [40]. It has also been reported that cytochrome c release can activate initiator caspase-9, which eventually activates executioner caspases like caspase-3 via cleavage [41]. We conclude that APNT plasma effectively promotes activation of caspases and a loss of MMP that results in the decline of cellular viability (**Figure 7**).

We also found that plasma treatment showed no or less effect on SNU80 cells. This anomalous behavior is due to the anaplastic condition of these cells. SNU80 is an anaplastic thyroid carcinoma (ATC) cell line obtained from Korean thyroid carcinoma patients, which divides rapidly and has little or no resemblance to normal cells. The mechanism of its carcinogenesis is unclear, and they are highly resistant to chemotherapy and radiotherapy. No effective therapeutic regimen has been identified for ATC. We are now focusing on novel therapeutic approaches to treat SNU80-like cell lines using various strategies with plasma treatment and will report on these experiments in the future.

Based upon our findings in this report, decreased viability of cancer cells strongly suggests that oxidative stress induced by plasma resulted in the observed cell death (**Figure 1**, **2**). GSH, one of the major redox state regulators, is known to be involved in elimination of ROS [42]. To test this hypothesis, we analyzed antioxidant markers and showed decreased levels of both GSH and NADPH. That revealed a compromised redox state that may be responsible for the cell death (**Figures 5**, **6** and **8**). Results from the current study emphasize the need for a deeper understanding of the role that plasma induced reactive oxygen species play in tumor suppression. Our study suggests that the failure of the antioxidant system to neutralize ROS is responsible for ROS elevation and cell death. Excess ROS and H_2_O_2_, which are not reduced by antioxidant enzymes inside the mitochondria, may also damage mitochondria and can cross the mitochondrial membrane into the cytosol and cause cell damage. These findings offer insight into the mechanism underlying APNT plasma-mediated apoptosis for future studies that may provide insight into methods to develop better plasma-therapeutic approaches that are selective for various types of cancer cells.
